# Compartmentalization of lipid peroxidation in sepsis by multidrug-resistant gram-negative bacteria: experimental and clinical evidence

**DOI:** 10.1186/cc11930

**Published:** 2013-01-16

**Authors:** Chryssoula Toufekoula, Vassileios Papadakis, Thomas Tsaganos, Christina Routsi, Stylianos E Orfanos, Anastasia Kotanidou, Dionyssia-Pinelopi Carrer, Maria Raftogiannis, Fotini Baziaka, Evangelos J Giamarellos-Bourboulis

**Affiliations:** 1 4th Department of Internal Medicine, University of Athens, Medical School, 12462 Athens, Greece; 2 1st Department of Critical Care Medicine, University of Athens, Medical School, 10676 Athens, Greece; 3 2nd Department of Critical Care Medicine, University of Athens, Medical School, 12462 Athens, Greece; 4 Integrated Research and Treatment Center, Center for Sepsis Control and Care (CSCC), Jena University Hospital, 07747 Jena, Germany

## Abstract

**Introduction:**

Recent evidence suggests a link between excess lipid peroxidation and specific organ failures in sepsis. No study has been performed in sepsis by multidrug-resistant (MDR) Gram-negative bacteria.

**Methods:**

Lethal sepsis was induced in rats by the intraperitoneal injection of one MDR isolate of *Pseudomonas aeruginosa*. Produced malondialdehyde (MDA) was measured in tissues 5 hours after bacterial challenge with the thiobarbiturate assay followed by high-performance liquid chromatography (HPLC) analysis. Results were compared with those from a cohort of patients with ventilator-associated pneumonia (VAP) and sepsis by MDR Gram-negative bacteria. More precisely, serum MDA was measured on 7 consecutive days, and it was correlated with clinical characteristics.

**Results:**

MDA of septic rats was greater in the liver, spleen, and aortic wall, and it was lower in the right kidney compared with sham operated-on animals. Findings were confirmed by the studied cohort. Circulating MDA was greater in patients with hepatic dysfunction and acute respiratory distress syndrome (ARDS) compared with patients without any organ failures. The opposite was found for patients with acute renal dysfunction. No differences were found between patients with ARDS without or with cardiovascular (CV) failure and patients without any organ failure. Serial measurements of MDA in serum of patients indicated that levels of MDA were greater in survivors of hepatic dysfunction and ARDS and lower in survivors of acute renal dysfunction.

**Conclusions:**

Animal findings and results of human sepsis are complementary, and they suggest a compartmentalization of lipid peroxidation in systemic infections by MDR gram-negative bacteria.

## Introduction

Oxidative stress results from an imbalance between production of reactive oxygen and nitrogen species (ROS and RNS) and endogenous antioxidant defense mechanisms [1]. A growing body of evidence suggests that many of the effects of cellular dysfunction under oxidative stress are mediated by products of nonenzymatic reactions, such as the peroxidative degradation of polyunsaturated fatty acids. Aldehyde molecules generated during lipid peroxidation are considered ultimate mediators of toxic effects elicited by oxidative stress occurring in biologic material. Malondialdehyde (MDA) is the most abundant lipid peroxidation-specific aldehyde [2].

The process of lipid peroxidation accompanied by excess production of MDA has been well documented both in experimental studies with cells infected by specific pathogens [3] and in systemic infections complicated by sepsis and leading to death [4,5]. Available evidence from studies in humans evokes the impression that lipid peroxidation is a harmful event ubiquitously accompanying all cases of systemic inflammation and sepsis to such an extent that circulating MDA may be conceived of as a biomarker of unfavorable prognosis [4-6]. Surprisingly, a recent study in patients with severe sepsis (that is, sepsis complicated by organ failure) showed that MDA is increased in the circulation of patients with hepatic failure and with renal failure but not in the circulation of patients with respiratory failure and circulatory failure [7]. These results were generated in a cohort of 50 sepsis patients and required further confirmation. However, they suggest that lipid peroxidation is not a ubiquitous phenomenon, and that it may be implicated in specific organ failures in a compartmentalized fashion.

Many cases of sepsis develop as complications of hospitalization in the intensive care unit (ICU). In such cases, the offending pathogens are often multidrug resistant (MDR), and they impose considerable difficulties in management because the available antimicrobial agents are limited [8]. The only solution to this emerging problem is the in-depth understanding of the pathogenesis behind sepsis caused by MDR bacteria. At this point, it should be underscored that the studied models of sepsis use either purified PAMPs (pathogen-associated molecular patterns) or peritonitis after CLP (cecal ligation and puncture) as a stimulus. MDR bacteria are considered to stimulate host responses in a fashion similar to that of community-acquired susceptible bacteria. However, much ambiguity exists whether MDR gram-negative isolates possess different virulence characteristics from susceptible gram-negative isolates. This is very difficult to conclude from a clinical study in which inappropriate antimicrobial therapy is the driver for unfavorable outcomes after infection with MDR bacteria and does not allow focus on precise virulence characteristics [9]. In a recent retrospective study, the physical course of 29 patients infected with carbapenem-resistant Enterobacteriaceae was compared with that of 29 well-matched patients infected with carbapenem-susceptible Enterobacteriaceae; in-hospital mortalities were 30.8% and 7.4%, respectively (*P *= 0.04) [10]. Similar results were drawn from a retrospective study of our group on the outcomes of 243 patients; however when patients with severe sepsis were analysed separately, it was found that severe sepsis caused by MDR bacteria was accompanied by prolonged survival compared with severe sepsis caused by susceptible bacteria [11]. As a consequence, it may be hypothesized that the pathogenesis of infections by MDR bacteria may have specific peculiarities.

**Figure 1:**
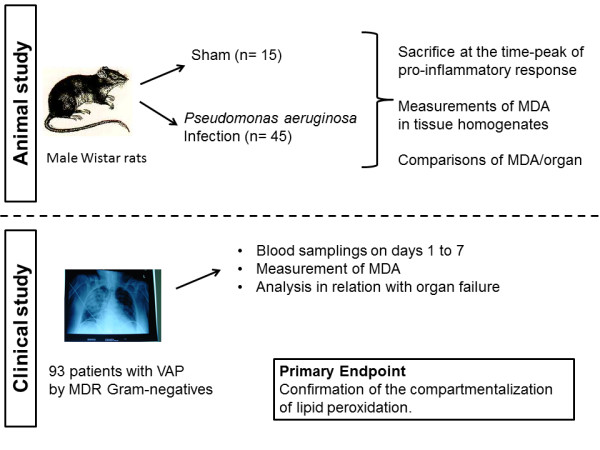
**Study flow chart**. MDA, malondialdehyde; MDR, multidrug resistant; VAP, ventilator-associated pneumonia. The chest radiograph is of a patient enrolled in the study cohort, with permission.

It is thus evident that in-depth understanding is required on the process of lipid peroxidation for infections caused by MDR bacteria. This is based on (a) the compartmentalization of the process of lipid peroxidation for the generation of organ failures coming from one cohort of patients with severe sepsis [7]; and (b) different clinical characteristics of severe sepsis elicited by MDR bacteria from those by susceptible bacteria [11]. To this end, a dual approach was used: first, we studied lipid peroxidation in tissues in a lethal sepsis model with MDR *Pseudomonas aeruginosa*, and then we tried to confirm findings of circulating MDA in patients with specific organ failures after ventilator-associated pneumonia (VAP) from MDR gram-negative bacteria (Figure 1).

## Materials and methods

### Animal study

In total, 60 male Wistar rats were studied. Their mean ± SD weight was 253.4 ± 22.4 g. The study received permission from the Veterinary Directorate of the Perfecture of Athens, according to the Greek legislation, in conformance to the Council Directive of the European Community. Rats were housed in metal cages and had access to tap water and standard balanced chow *ad libitum*. Temperature ranged between 18°C and 22°C; relative humidity, between 55% and 65%; and the light/dark cycle was 6 AM to 6 PM. One multidrug-resistant (MDR) isolate of *P. aeruginosa *from the blood of a patient with severe sepsis was applied. Minimal inhibitory concentrations (MICs) of piperacillin/tazobactam, ceftazidime, imipenem, meropenem, ciprofloxacin, and amikacin, determined with a microdilution technique, were 256/4, 256, 128, 64, 512, and 512 μg/ml, respectively. The isolate was stored as multiple aliquots in skim milk (Oxoid Ltd, London, UK) under -70°C. One aliquot was removed from the refrigerator before each experiment. Single colonies were incubated at 37°C in 10 ml of Mueller-Hinton broth (Oxoid Ltd) for 8 hours to yield a log-phase inoculum that was applied for bacterial challenge.

Animals were divided into two groups for treatment, as follows:

• Group A (*n *= 15), sham-operated; injected intraperitoneally with 1 ml of Mueller-Hinton; and

• Group B (*n *= 45), animals injected intraperitoneally with 1 ml of the prepared inoculum of the test isolate yielding a challenge of 5 × 10^6 ^cfu/rat.

Survival was recorded at 12-hour intervals for four animals of group A and for 10 animals of group B. To identify the peak time point of lipid peroxidation, animals of group B were killed at serial time intervals with six rats per interval. At every time of sacrifice, a midline abdominal incision was performed. Intestines were displaced to the left, and the inferior vena cava was recognized and punctured with a 19-gauge needle. Five milliliters of blood was collected into pyrogen-free syringes and applied for the measurement of tumor necrosis factor-alpha (TNF-α) and of malondialdehyde (MDA). Animals were then killed with an intramuscular injection of pentothal.

These experiments were repeated in six animals per group at the defined peak time point of serum MDA. Under sterile conditions, segments from the liver, spleen, lower lobe of the right lung, the heart, the right kidney and of the abdominal aorta were taken and placed into separate sterile plastic containers. In these experiments, 5 ml of blood was sampled after venipuncture of the lower vena cava under aseptic conditions and collected into EDTA-containing tubes (Becton Dickinson, Cockeysville, MD, USA) for the measurement with flow cytometry of an oxidative burst on neutrophils. Tissue segments were weighed and homogenized by using 1 ml of sterile phosphate-buffered saline (PBS; pH 7.2). For the measurement of tissue bacterial outgrowth, aliquots of 0.1 ml of the homogenates were diluted 1:10 into sterile 0.9% NaCl for four consecutive times. Aliquots of 0.1 ml of each dilution were plated onto McConkey agar and incubated at 35°C for 3 days. The number of viable colonies was counted by multiplying with the appropriate dilution factor. The lower limit of detection was 10 cfu/g. The number of viable cells in tissues was expressed by log_10 _values.

Lipid peroxidation was estimated in serum and in tissue homogenates by the concentration of MDA, as already described [12]. In brief, a 0.1-ml aliquot of each sample was mixed with 0.9 ml of trichloroacetic acid 20% (Merck, Darmstadt, Germany) and centrifuged at 12,000 *g *and 4°C for 10 minutes. The supernatant was removed and incubated with 2 ml of thiobarbituric acid, 0.2% (Merck) for 60 minutes at 90°C. After centrifugation, a volume of 10 μl of the supernatant was injected into a high-performance liquid chromatography system (HPLC; Agilent 1100 Series, Waldbronn, Germany) with the following characteristics of elution: Zorbax Eclipse XDB-C18 (4.6 × 150 mm, 5 μm) column under 37°C; mobile phase consisting of 50 m*M *K_3_PO_4 _(pH 6.8) buffer, and methanol 99% at a 60:40 ratio with a flow rate of 1 ml/min, fluorometric detection with signals of excitation at 515 nm and emission at 535 nm. The retention time of MDA was 3.5 minutes, and it was estimated in millimoles per milliliter with a standard curve created with 1,1,3,3-tetramethoxy-propane (Merck). All determinations were performed in duplicate. The lowest limit of detection was 0.1 mmol/ml, and the interday variation of the assay was 1.01%. Tissue concentrations of MDA were expressed as millimoles per gram.

Concentrations of serum TNF-α were measured in duplicate with an enzyme immunoassay (Diaclone, Marseille, France). The lower limit of detection was 12 pg/ml.

For the measurement of the oxidative burst on neutrophils, 0.1 ml of EDTA-blood was aliquoted into separate sterile tubes. A volume of 20 μl of phorbol-myristate (PMA, Sigma, St. Louis, MO, USA; final concentration, 10 μ*Μ*) was added in the second tube, and the first tube was left untreated. Both tubes were incubated for 10 minutes at 37°C in a water bath. Then 10 μl of DHR (dihydrorhodamine; Sigma, final concentration 100 μ*Μ*) was added. After 10 minutes of incubation at 37°C in a water bath, 1 ml of VersaLyse was added in both tubes for the lysis of red blood cells (Immunotech, Marseille, France). After 15 minutes of incubation in the dark, tubes were analyzed with the FC500 counter (Beckman Coulter, Miami, FL, USA). Oxidative burst was expressed as the mean fluorescence intensity (MFI) of DHR on granulocytes after gating by the characteristic FS/SS scattering by using PMA-untreated cells as negative controls.

### Clinical study

Serum samples were collected daily on the first 7 days from 93 patients enrolled in the placebo arm of a double-blind, randomized, placebo-controlled clinical trial for the management of VAP and sepsis [13]. The study was performed during the period from June 2004 to November 2005, and patients were hospitalized in the Department of Critical Care Medicine of the "Evangelismos" General Hospital, in the 2^nd ^Department of Critical Care Medicine of the "ATTIKON" University Hospital of Athens and in the 4^th ^Department of Internal Medicine of the "ATTIKON" University Hospital of Athens. The study was conducted after approval of the Ethics Committees of Evangelismos General Hospital and of ATTIKON University Hospital and after written informed consent by first-degree relatives. Patients were divided into those with hepatic dysfunction, acute respiratory distress syndrome (ARDS), acute renal dysfunction, and cardiovascular failure (CV) by standard definitions [14]. Three microliters of blood was sampled for 7 consecutive days after venipuncture of one forearm vein under aseptic conditions and collected into pyrogen-free tubes (Vacutainer, Becton Dickinson). The tubes were immediately centrifuged, and serum was kept refrigerated at -70°C until assayed. Concentrations of MDA were measured in serum, as described earlier.

### Study end points

The primary study end point was the existence of compartmentalization of lipid peroxidation in relation to the specific failing organs. This was assessed in an experimental model in rodents, and it was tested for confirmation by the measurement of circulating MDA in patients with sepsis.

The secondary study end point was the correlation between circulating MDA and final outcome in relation to the underlying organ failure.

### Statistical analysis

For animal experiments, survival was estimated with Kaplan-Meier analysis. Concentrations of MDA and TNF-α were expressed by their means ± SEM. Comparisons of concentrations of MDA and of TNF-α in serum between serial times of sampling were done with one-way analysis of variance (ANOVA) with *post hoc *Bonferroni corrections. Comparisons of tissue MDA between groups A and B were performed with the Mann-Whitney *U *test. Correlations between tissue MDA and bacterial growth were according to Spearman's rank of order.

For the clinical study, the MDA of the first day in serum was expressed as median and 95% confidence intervals. Comparisons between patients with organ failures and those without any organ failures were done with the Mann-Whitney *U *test, analyzing also for outliers. To demonstrate the impact of lipid peroxidation in the pathogenesis of liver dysfunction and of renal dysfunction, all patients with acute hepatic dysfunction and all patients with acute renal dysfunction were grouped together as having hepatic dysfunction and renal dysfunction, respectively, whether or not they had other organ failures. MDAs of consecutive days were expressed as mean ± SEM. Comparisons between survivors and nonsurvivors were done with the Mann-Whitney *U *test. For all comparisons, any value of *P *< 0.05 after adjustment for multiple comparisons with Bonferroni was considered significant.

## Results

### Animal infection model

**Figure 2:**
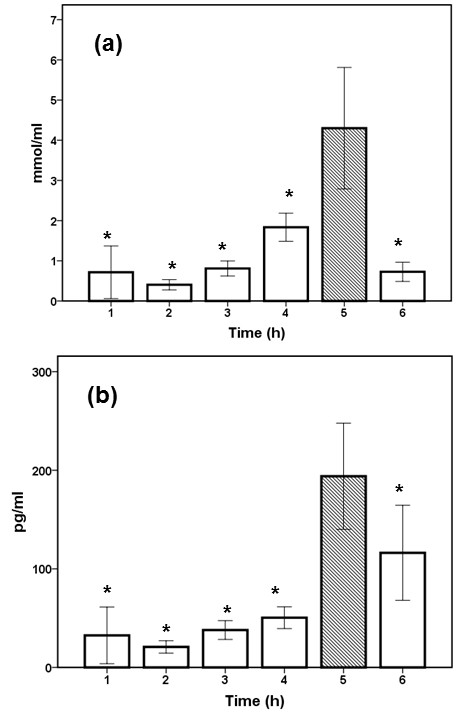
**Serial concentrations of tumor-necrosis factor-α (TNF-α) and malondialdehyde (MDA) after bacterial challenge**. Experimental sepsis was induced in rats after the intraperitoneal injection of one multidrug-resistant isolate of *Pseudomonas aeruginosa*. At the indicated time intervals after challenge (*n *= 6 per time), rats were killed, and concentrations of MDA **(a) **and of TNF-α **(b) **were measured in serum. *P < 0.05 compared with the 5-hour interval after adjustment for multiple comparisons.

Mortality of the studied sepsis model by MDR *P. aeruginosa *was 50% at 12 hours after bacterial challenge and 100% at 36 hours after bacterial challenge. None of the sham-operated-rats died. Sacrifice experiments conducted at serial time intervals after bacterial challenge found the peak of serum MDA at 5 hours (Figure 2). This coexisted with the peak of the proinflammatory response as indexed by serum TNF-α. Based on this finding, it was decided that sacrifice experiments with measurement of tissue lipid peroxidation should be repeated in rats killed at 5 hours after challenge with the test isolate.

**Figure 3:**
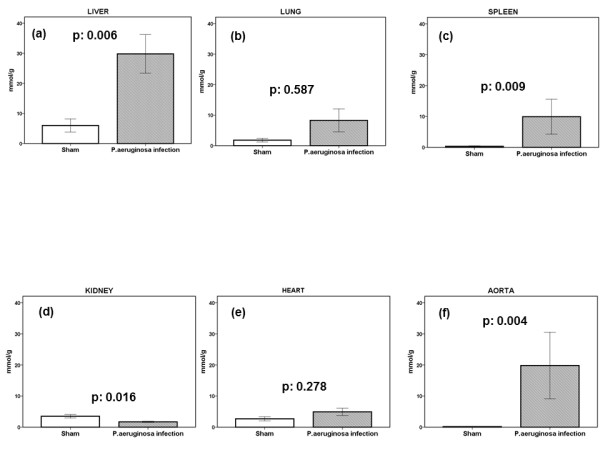
**Tissue concentrations of MDA after bacterial challenge**. Experimental sepsis was induced in rats after the intraperitoneal injection of 2 × 10^7 ^cfu/animal of one multidrug-resistant isolate of *Pseudomonas aeruginosa*. Five hours after challenge (*n *= 6 per group), rats were killed, and concentrations of malondialdehyde (MDA) were measured in the tissue homogenates of liver **(a)**, lung **(b)**, spleen **(c)**, right kidney **(d)**, heart **(e)**, and aorta **(f)**. *P *values compared with sham-operated rats are shown.

These experiments showed that MDA was particularly elevated in liver, in spleen, and in the aortic wall (Figure 3); however, MDA was significantly reduced in the right kidney. Paired comparisons by the Wilcoxon signed-rank test showed that MDA in the liver was greater than that in the lung (*P *= 0.016), in the spleen (*P *= 0.041), in the heart (*P *= 0.004), but not in the aortic wall (*P *= 0.116). At the time of death, tissues of sham-operated-on rats were sterile. However, mean ± SEM counts of the injected isolate in the liver were log_10 _3.95 ± 0.63 cfu/g; in the spleen, log_10 _4.29 ± 0.71 cfu/g; in the lung, log_10 _3.07 ± 0.76 cfu/g; in the heart, log_10 _2.55 ± 0.58 cfu/g; and in the aortic wall, log_10 _3.88 ± 0.67 cfu/g. Paired comparisons did not show any difference between the bacterial growth of the liver compared with the other tissues. No significant correlation could be found between MDA and bacterial growth in tissues (correlation for liver, r_est_, -0.273; *P *= 0.417; for lung, r_est_, +0.040; *P *= 0.841; for spleen, r_s_, -0.445; *P *= 0.170; for right kidney, r_s_, +0.080, *P *= 0.815; for heart, r_s_: -0.516, *P *= 0.295; and for aorta, r_s_: +0.123, *P = *0.715; data not shown).

**Figure 4:**
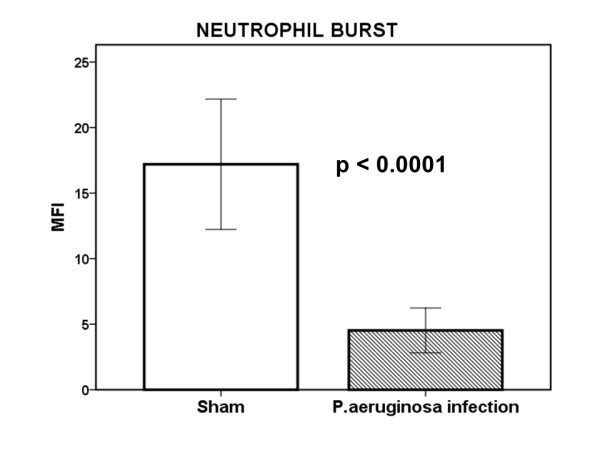
**Oxidative burst of circulating neutrophils**. Experimental sepsis was induced in rats after the intraperitoneal injection of one multidrug-resistant isolate of *Pseudomonas aeruginosa*. Five hours after challenge (*n *= 6 per time), rats were killed, and oxidative burst on neutrophils was measured as the expression of dihydrorhodamine (DHR) on cell membranes after flow-cytometric analysis. P value compared with sham-operated rats is shown.

At the time of death, the neutrophil burst was defective in circulating neutrophils of infected rats (Figure 4).

### Clinical study

Results of the animal experiments prompted us to hypothesize that a compartmentalization of the oxidant status takes place in sepsis. To investigate whether this is also the situation for human sepsis, we measured circulating MDA in 93 patients with sepsis, making the hypothesis that serum MDA should differ between patients as a reflection of the failing organ. These patients were enrolled in the placebo arm of a randomized trial previously published [13,15]. All had VAP, and the pathogens isolated in the quantitative tracheobronchial secretions were MDR species of *Acinetobacter baumannii*, of *Pseudomonas aeruginosa*, and of *Klebsiella pneumoniae*. As a consequence, this study cohort could be used to extrapolate animal findings because all patients had the same source of sepsis (that is, VAP), and they were infected with MDR bacteria. The demographic characteristics of these patients were described elsewhere [13].

**Figure 5:**
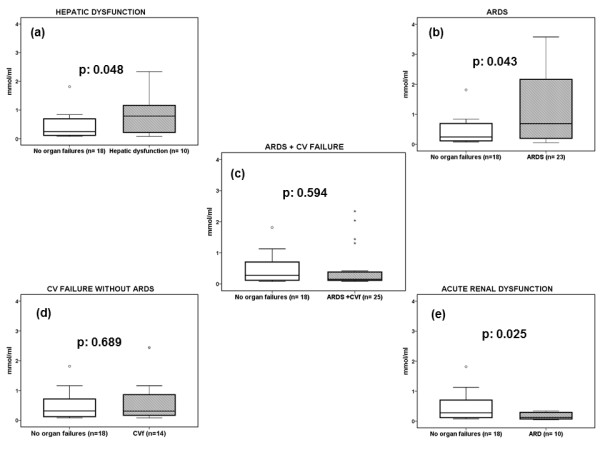
**Serum concentrations of MDA in patients with sepsis**. Concentrations of malondialdehyde (MDA) were measured on the first day of sepsis due to ventilator-associated pneumonia with multidrug-resistant gram-negative bacteria in 93 patients. Results are presented according to the types of failing organs: **(a) **patients with hepatic dysfunction versus patients without any organ failure; **(b) **patients with ARDS versus patients without any organ failure; **(c) **patients with ARDS and CV failure versus patients without any organ failure; **(d) **patients with CV failure without ARDS versus patients without any organ failure; and **(e) **patients with acute renal dysfunction versus patients without any organ failure). Circles denote outliers. *P *values reflect comparisons between patients without any organ failure and with the indicated organ failure. ARDS, acute respiratory distress syndrome; CV, cardiovascular.

Analysis of circulating MDA was done in relation to the type of failing organ. From these 93 patients, 18 did not have any organ failure; the mean ± SEM pO_2_/FiO_2 _of the first day of sampling of these patients was 338.9 ± 34.8 mm Hg. This pO_2_/FiO_2 _ratio showed that they did not have acute lung injury, and they could be used as appropriate comparators for this analysis. Another 10 patients had hepatic dysfunction, 23 had only ARDS; 25 had ARDS and CV failure; 14 had CV failure without ARDS; and 10 had acute renal dysfunction. Of the 10 patients with acute hepatic dysfunction, four also had other organ failures. Of the 10 patients with acute renal dysfunction, three also had other organ failures. Circulating concentrations of MDA are shown in Figure 5. It was obvious that circulating MDA was increased in the case of hepatic dysfunction and in the case of ARDS. In patients with both ARDS and CV failure or with CV failure without ARDS, MDA did not increase. Circulating MDA was lower in patients with acute renal dysfunction than in patients without organ failures and corroborated the findings of the experimental infection model in rats.

**Figure 6:**
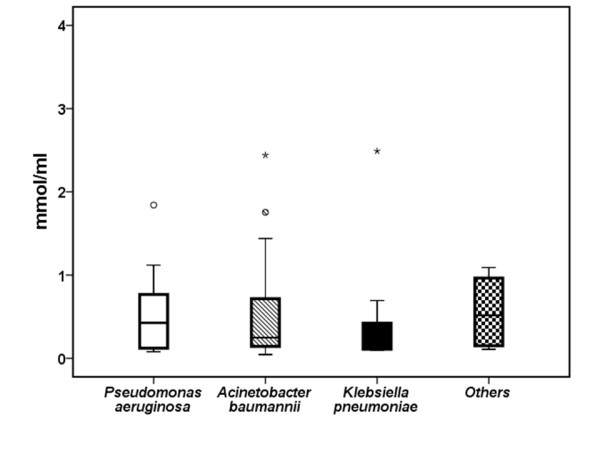
**Serum concentrations of MDA in patients with sepsis**. Concentrations of malondialdehyde (MDA) were measured on the first day of sepsis due to ventilator-associated pneumonia with multidrug-resistant gram-negative bacteria in 93 patients. Results are presented according to the type of implicated pathogen. Circles denote outliers, and asterisks denote extremes.

Of the 93 enrolled patients, the implicated pathogen was isolated from quantitative tracheobronchial secretions at a quantity > 10^5 ^cfu/ml from 60 patients. More precisely, *Pseudomonas aeruginosa *was isolated from 10 patients, *Acinetobacter baumannii *from 38 patients, *Klebsiella pneumoniae *from six patients, and other gram-negative species from six patients. Microbiology documentation failed in 30 patients. Serum MDA in relation with the microbiology of VAP is shown in Figure 6. No differences were found in relation to the underlying pathogen (*P *= 0.925).

**Figure 7:**
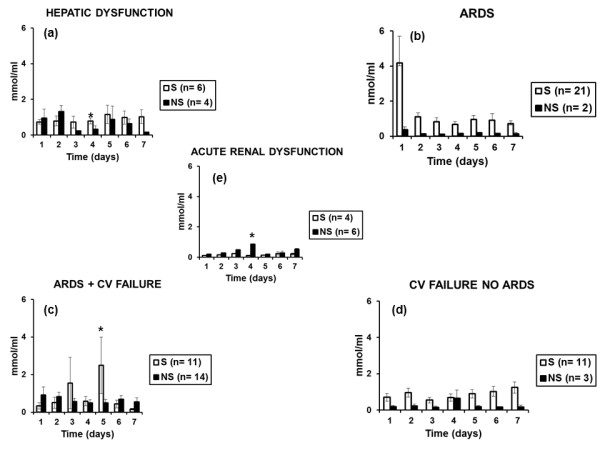
**Follow-up measurements of MDA of serum in patients with sepsis**. Concentrations of malondialdehyde (MDA) were measured for 7 consecutive days in patients with ventilator-associated pneumonia with multidrug-resistant gram-negative bacteria and sepsis. Results are presented according to the type of failing organ: **(a) **patients with hepatic dysfunction; **(b) **only ARDS patients; **(c) **patients with ARDS and CV failure; **(d) **patients with CV failure without ARDS; and **(e) **patients with acute renal dysfunction, and separately for survivors (S) and nonsurvivors (NS). *P < 0.05 between S and NS.

Follow-up measurements in survivors and nonsurvivors from organ failures are shown in Figure 7. Time-kinetics of MDA differed in relation to the type of failing organ(s). More precisely, serum MDA was greater in survivors from hepatic dysfunction on day 4 and in survivors from ARDS and CV failure on day 5. Serum MDA was also lower on day 4 among survivors from acute renal dysfunction. Similar statistical comparisons could not be done for patients with single ARDS, because the number of nonsurvivors was low (*n *= 2).

No statistical correlation could be found between serum MDA and survival time for any of these subgroups of patients (data not shown).

## Discussion

Excess formation of lipid peroxides in serum accompanied by consumption of the total antioxidant capacity of blood has been demonstrated to take place over the time-course of experimental bacteremia by MDR *P. aeruginosa *in rabbits [16]. In the present study, in-depth investigation in an animal model of lethal sepsis by the same MDR species revealed that lipid peroxidation was highly compartmentalized. Excess formation of MDA took place in the liver, in the spleen, and in the aortic wall, whereas that was not the case for the lung and for the heart. On the contrary, MDA was decreased in the kidneys. It should be overemphasized that, at the time of animal death, when lipid peroxidation differed considerably within tissues, the oxidative burst of circulating neutrophils was defective. This finding is in accordance with reports that the function of neutrophils becomes defective in severe sepsis in exactly the same way that immunoparalysis succumbs in the constellation of infection-induced organ failures [17]. However, this finding may also reflect, at least in part, that in the studied setting of widespread systemic infection by MDR *P. aeruginosa*, oxidative stress fails in circulating neutrophils, but it is highly generated in tissues, probably as a result of infiltration by neutrophils.

These findings corroborate many of the findings of other investigators, but they also present discrepancies with other studies in rodents. In these studies, investigators used either purified bacterial lipopolysaccharide (LPS) or CLP to stimulate host defenses. MDA in liver homogenates had been increased in rats killed 6 hours after the intraperitoneal injection of LPS [18]. When a model of CLP was studied in rats, downregulation of the expression of genes coupled with mitochondrial function was found [19]. This is consistent with mitochondrial uncoupling from excess ROS formation and corroborates the presented findings. However, the described lack of excess lipid peroxidation in the heart muscle after challenge by MDR *P. aeruginosa *herein is in disagreement with two recent publications. In the first publication in mice [20], excess ROS formation was found in the myocardium 48 hours after CLP. In the present study, animal killing was done at 5 hours (that is, earlier than the mouse CLP model), and this may explain the discrepancy. In the second publication, excess formation of ROS was found in the heart tissue as early as 3 hours after bacterial challenge. However, this was a completely different model of sepsis induced after aspiration challenge with *Streptococcus pneumoniae *[21].

Infections by MDR bacteria emerge as a serious problem in the hospital environment. They colonize patients after long-term hospital stay, and they are the cause of severe sepsis after ICU admission. Available choices for antimicrobial therapy become more and more limited, creating major hurdles. Limitations of management shed light on interventions on the host response. Antioxidant supplementation against excess formation of ROS and RNS belongs to these interventions [22].

Animal sepsis models offer the advantage of well-defined infection settings and of clear-cut approaches. The human situation is far more difficult; comorbid conditions play a pivotal role, and only indirect simulations can be done. Trying to validate the generated animal data in human sepsis, it was decided that one hospital-acquired infection by MDR gram-negative bacteria should be studied. To this end, a cohort of patients with VAP by MDR gram-negative species was studied [13]. This cohort proved to be an adequate equivalent of clinical sepsis by MDR bacteria, because circulating MDA did not differ in relation to the implicated bacterial species.

Circulating MDA was correlated with the types of failing organs. Results indicated that MDA was particularly elevated in specific organ failures compared with patients with none organ failure corroborating the animal data for compartmentalization of lipid peroxidation. In a recent study [7], concentrations of the lipid metabolites F2-isoprostane and isoflurane were measured in 50 patients with severe sepsis. These metabolites were considered an indirect index of lipid peroxidation. Measured concentrations were greater in patients who developed hepatic failure compared with those who did not. Contrary to the presented clinical findings, F2-isoprostane and isoflurane were greater in the event of renal failure. In the same study of 50 patients with severe sepsis [7], levels of lipid metabolites remained unchanged in the event of ARDS and of CV failure. Many studies have provided evidence for excess lipid formation in the event of acute lung injury and of ARDS [23]. This is probably related to the high infiltration of the lung by neutrophils that overproduce ROS. Our study indicated that ARDS ensuing in the absence of CV failure is accompanied by excess lipid peroxidation. However, when ARDS is accompanied by CV failure, excess circulating MDA ceases to exist. The study of the levels of F2-isoprostane and isoflurane in severe sepsis did not study separately patients with ARDS without CV failure from patients with both ARDS and CV failure.

Many of the conducted clinical studies on the formation of ROS in human sepsis have shown that excess lipid peroxidation is an index of unfavorable outcome [5,6,23]. Findings of the present study suggest that kinetics of MDA in serum differ between survivors and nonsurvivors, depending on the type of organ failure. More precisely, MDA is increased over the disease course of survivors of hepatic dysfunction and of ARDS. On the contrary, it is decreased over the time course of survivors of acute renal dysfunction. The results of the current study do not provide mechanistic data to explain the findings, and only assumptions can be made. Regarding the increase of serum MDA within nonsurvivors of acute renal dysfunction, it may be hypothesized that although lipid peroxidation in not the principal mechanism for acute renal dysfunction, it participates in the progression toward unfavorable outcome. Furthermore, different patterns of the kinetics of serum MDA are shown in survivors and nonsurvivors of CV failure with and without ARDS. Taking into consideration the respective patterns in patients only with ARDS, it may be postulated that overall, serum MDA is elevated in the event of ARDS; this is decreased on addition of CV failure and further decreased in those only with CV failure.

Animal sepsis with MDR *P. aeruginosa *was accompanied by a defective oxidative burst of circulating neutrophils. However, tissue MDA of the spleen was increased. These findings are in accordance with the decrease in serum activity of myeloperoxidase described in patients with septic shock [24]. It may thus be hypothesized that in the event of severe sepsis and organ failures, circulating neutrophils are defective in efficient oxidative bursts and phagocytosis, whereas these same functions are upregulated in peripheral organs like the spleen and the lung.

The complementary findings between the described animal model and human sepsis are highly indicative of an existing compartmentalization of lipid peroxidation in systemic infections with MDR gram-negative bacteria. It should be underscored that such a study design is not devoid of limitations. The major limitation is the time course until the start of one organ failure, which is determined by the investigators of an animal study, in contrast to the clinical situation in which this is not possible to be defined with precision. However, the described compartmentalization may explain, at least in part, the contradictory findings of clinical studies with antioxidant supplementation in severe sepsis [23].

Results of the present study can be used to select the populations of patients at severe sepsis that may benefit from antioxidant agents, with emphasis on patients with hospital-acquired sepsis with MDR gram-negative species. Although it cannot be precluded that lipid peroxidation may be stimulated in a similar mechanism by gram-negative bacteria and by gram-positive cocci, the present results are limited on gram-negative sepsis, particularly because gram-negative species emerged as the main pathogens of the studied cohort.

## Conclusions

These findings coming from an experimental infection model that are confirmed from a patient cohort clearly indicate that, during systemic infections, lipid peroxidation is characterized by high compartmentalization. The phenomenon is considerably upregulated in the event of ARDS and hepatic dysfunction and downregulated in the event of acute renal dysfunction.

## Key messages

• High compartmentalization of lipid peroxidation exists in experimental *P. aeruginosa *infection

• Increased levels are found in liver, spleen, and aorta, and decreased levels are found in kidney.

• Results are confirmed in a cohort of patients with gram-negative sepsis

• Levels of MDA are increased in hepatic dysfunction and ARDS

• Levels of MDA are decreased in renal dysfunction and remain unchanged in cardiovascular failure

## Abbreviations

ARDS: acute respiratory distress syndrome; CLP: cecal ligation and puncture; CV: cardiovascular; DHR: dihydrorhodamine; LPS: lipopolysaccharide; HPLC: high-performance liquid chromatography; MDA: malondialdehyde; MDR: multidrug resistant; PMA: phorbol-myristate acetate; ROS: reactive oxygen species; TNF-α: tumor necrosis factor-alpha; VAP: ventilator-associated pneumonia.

## Competing interests

The authors declare that they have no competing interests.

## Authors' contributions

CT performed measurements of MDA, drafted the manuscript, and agreed to the final submitted version. VP, TT, and DPC performed animal experiments, drafted the manuscript, and agreed to the final submitted version. CR, SEO, AK, MR, and FB enrolled patients, collected serum samples and clinical data, drafted the manuscript, and agreed to the final submitted version. EJGB designed the study, wrote the manuscript, and agreed to the final submitted version.
